# Cat Coat Color, Personality Traits and the Cat-Owner Relationship Scale: A Study with Cat Owners in Mexico

**DOI:** 10.3390/ani12081030

**Published:** 2022-04-15

**Authors:** Mónica Teresa González-Ramírez, René Landero-Hernández

**Affiliations:** School of Psychology, Autonomous University of Nuevo León, San Nicolás de los Garza C.P. 64455, Mexico; rene.landerohr@uanl.edu.mx

**Keywords:** cat-owner interaction, cat personality traits, coat color

## Abstract

**Simple Summary:**

Studies regarding the cat-owner bond are quite rare, and several aspects like personality trait differences in cats related to coat color and the cat-owner relationship require more research. With that purpose, we apply a survey to 211 cat-owners from Mexico. Owners perceived their cats as being bold and friendly. Gray cats had the highest score for being as shy, aloof and intolerant, while orange cats had the highest scores for being trainable, friendly and calm. Tabbies the highest for bold and active, tricolor cats for stubborn, and bicolor cats for tolerant. Higher cat-owner interaction was related with an active and friendly personality and with lower score of aloofness. Higher emotional closeness was related with an active, bold and friendly personality, and higher perceived cost was related with lower score of boldness.

**Abstract:**

Studies regarding the cat-owner bond are quite rare, and several aspects merit more research, including personality trait differences in cats related to coat color and the cat-owner relationship. The objectives of the study were to describe, from the perspective of their owners, the personality traits of cats based on their coat colors and to evaluate the relationships among the Cat Owner Relationship Scale (CORS), its subscales and the traits of cats. Therefore, the CORS was translated into Spanish, and its psychometric properties were assessed. For the personality traits of cats, participants answered a 7-point Likert scale indicating the extent to which they agreed with the following characteristics in describing their cats: active, aloof, bold, calm, friendly, intolerant, shy, stubborn, tolerant and trainable. 211 cat owners living in Mexico participated. Owners perceived their cats as being bold and friendly. Gray cats had the highest score for being as shy, aloof and intolerant, while orange cats had the highest scores for being trainable, friendly and calm. Tabbies the highest for bold and active, tricolor cats for stubborn, and bicolor cats for tolerant. The 3 CORS subscales had adequate psychometric properties when evaluated separately. Cat-owner interaction was positively correlated with an active and friendly personality and negatively correlated with aloofness. Emotional closeness was positively correlated with an active, bold and friendly personality, and perceived cost was negatively correlated with boldness.

## 1. Introduction

Cats, dogs and other species are considered companion animals [1]. The preference for cats in households has recently increased [2], even surpassing preference for dogs in some countries [3], and in Europe, cats are the most common pet [4]. However, some years ago, Bernstein [3] indicated that studies regarding the cat-owner bond were still quite rare and that many aspects regarding cats and the cat-owner relationship required further study. Recently, Ines et al. [5] asserted that little is known about the emotional relationships that cats form with humans. One issue requiring more research is personality trait differences in cats based on their coat colors. Stelow et al. [6] suggested that people commonly believe that calico cats are “crazy”, black cats are “wild and unpredictable”, and orange cats are “friendly”.

The association between physical appearance and personality in mammals has been investigated in different species. In his famous research on fox domestication, Belyaev [7] concluded that physical differences were due to prolonged selection for a tame genotype. Díaz Videla [8] mentioned that specific phenotypes, e.g., coat color, could be considered indicators of personality traits in dogs. When Díaz Videla performed a systematic review studying black dog syndrome, he only found 18 studies that met his inclusion criteria for investigating personality traits associated with coat color in dogs, demonstrating the scarcity of studies in this regard [8].

The hypothetical relationship between coat color and personality traits is based on the fact that the pigment melanin shares a synthesis pathway with a group of catecholamines and neurotransmitters [9], such as dopamine, which may lead to associations between pigmentation and personality traits [8]. Although such associations have rarely been studied in domestic cats [10], Stelow et al. [6] cited a series of studies indicating that a cat’s personality is stable from kittenhood, and that a genetic component is apparently involved; for example, Siamese cats have been reported to be more demanding compared with other breeds.

Delgado et al. [10] reported that the few studies related to cat color and personality show mixed results. In their research, they asked about the degree to which people felt that the color of a cat reflected specific personality traits and found that people believe that orange cats are the friendliest, tricolor cats the most intolerant, and white and tricolor cats the most aloof. They found no significant differences in terms of stubbornness. In the same study, white cats were reported to be perceived as less active, shyer and calmer than other cats. In another study, Stelow et al. [6] reported that cat owners believe that bicolor cats are more frequently aggressive toward humans.

The impact of this type of research can be reflected in cat adoption and abandonment. People report that when selecting a cat to adopt, the cat’s personality is a more important factor than its color. Nevertheless, adoption decisions are influenced by the color of a cat when people have beliefs about the relationship between color and personality [10]. Thus, although adopting people say that color is not relevant and that they consider how playful cats are and how willing they are to interact with people black cats usually remain in shelters longer before being adopted [11]. In addition, Carini et al. [12] found that coat color can be advantageous or disadvantageous with respect to adoption opportunities for cats in shelters.

Although Evans et al. [13] suggested that the features that humans consider when choosing a cat remain unclear, they cite studies mentioning that cats are often selected for their color (e.g., Robinson [14]). Evans et al. [13] added that the personality of a cat can predict owner satisfaction with the relationship and documented the important impact of pets, which are often thought of as family members, on the lives of their owners.

When pets are perceived as family members, they can serve as social resources that promote human health and well-being [15]. Thus, a good cat-owner relationship can reasonably be anticipated to generate benefits for the cat’s owner and motivate him or her to care for and ensure the cat’s quality of life; in contrast, a bad relationship may result in abuse, neglect or even abandonment [2].

Howell et al. [2] adapted the Monash Dog-Owner Relationship Scale (MDORS), designed by Dwyer et al. [16] to evaluate the cat-owner relationship. This scale is known as the Cat Owner Relationship Scale (CORS) and is based on social exchange theory, which specifies that relationships are maintained only when the perceived cost and benefits are balanced or when the perceived benefits are greater than the costs of the relationship. The CORS includes 1 negative component of the relationship, perceived cost, and 2 components more oriented toward evaluating the relationship: interactions and perceived emotional closeness [2].

Based on the above information, the main objectives of the present study were to describe, from the perspective of their owners, the personality traits of cats based on their coat colors and to evaluate the relationship between the CORS subscales and the personality traits of cats. Accordingly, the CORS needed to be translated into Spanish and evaluated for its psychometric properties.

## 2. Materials and Methods

### 2.1. Participants

Cat owners living in Mexico participated in this study. Snowball sampling was used by asking participants to seek other people with cats to answer the questionnaire. An online system (SurveyMonkey.com accessed on 1 July 2019) was used. The survey link was posted on the author’s wall on Facebook, and contacts were asked to share it. No cat characteristics were used as inclusion criteria. The survey was closed after 2 weeks of no new participants. We received 235 questionnaires, incomplete ones were eliminated, resulting in a sample of 211 participants.

### 2.2. Instruments

The Cat Owner Relationship Scale (CORS), adapted by Howell et al. [2] from the MDORS [16] was used. The CORS consists of 26 items scored on a 5-point Likert-type scale and is divided into 3 subscales, with 6 items for the pet-owner interaction subscale, 11 items for the perceived emotional closeness subscale and 9 items for the perceived cost subscale. Howell et al. [2] reported adequate psychometric properties for the CORS. Considering that a version of the MDORS has been translated into Spanish for the Mexican population, with adequate psychometric properties [17], this translation was used for the present study, replacing the word dog with cat and using the back translation method for the items added by Howell et al. [2] for the CORS. The version used in this study is presented in Appendix A.

For the personality traits of cats, the research by Delgado et al. [10] was used as a reference. Using a 7-point Likert scale (ranging from totally agree to totally disagree), the participants were asked: from your perspective, how present are each of the following characteristics in your cat. Characteristics were: active, aloof, bold, calm, friendly, intolerant, shy, stubborn, tolerant and trainable (Appendix B). These 10 traits were selected by Delgado et al. [10] based on previous studies. To facilitate the comparison of personality traits between cats grouped by color, an index ranging from 0 to 100 was calculated based on the intragroup mean such that the extent to which a particular trait was evident in each color group could be determined.

The question addressing cat colorpoint was a close-ended question referring to the 5 basic colors described in the study by Delgado et al. [10] and those in the study by Stelow et al. [6], i.e., orange or yellow, tricolor, white, black, gray, bicolor and Siamese, with the addition of tabby, calico, tortoiseshell and other. Owners were also asked to describe the color of their cats if they felt that a description was necessary. Based on these responses, the colors were recoded using the following classification: white, orange, gray, black, bicolor, tricolor and tabby.

Participants also responded to some questions about demographics, including age, gender and marital status. Additionally, they reported the number of cats in their houses and whether they also had dogs. Participants were instructed to choose one of their cats if they had more than one when providing demographic information (age and sex) and completing the questionnaires described above. A pilot test was carried out to assess the understanding of the questionnaires.

### 2.3. Statistical Analysis

All statistical analyses were performed with IBM^®^ SPSS^®^ Statistics version 26 (IBM, Armonk, NY, USA). We began with a descriptive analysis of the variables using the Kolmogorov-Smirnov test to determine whether the data had a normal distribution. Because the data did not follow a normal distribution (*p* < 0.05), we used nonparametric tests, Spearman correlations and the Kruskal-Wallis test, and chi-squared test to evaluate cat color and sex association.

Also using IBM^®^ SPSS^®^ Statistics version 26, we conducted a simulation using weighted data technique to estimate the results that would be obtained in a larger sample. This technique gives cases specific weights, using simulated replication. With weighted data the sample size can be modified arithmetically. To Weight Cases, in SPSS, from the menus choose *Data*, then *Weight Cases by*, and a frequency variable should be selected. The values of the frequency variable are used as case weights. In this study, each participant was assigned a specific weight of 5, simulating that the sample and therefore the subgroups were 5 times larger.

For the analysis of the CORS, Cronbach’s alpha coefficient was used, and exploratory and confirmatory factor analyses were performed with SPSS Amos version 24 (Computer Program. Chicago: IBM SPSS) using the maximum likelihood method. The following goodness of fit statistics were considered: the chi-squared test (X^2^/df) a good fit is indicated by a small ratio, and values less than 3 indicate a good fit. The goodness-of-fit index (GFI) developed by Jöreskog and Sörbom [18], a value of 1 indicates a perfect fit; the adjusted goodness-of-fit index (AGFI) developed by the same authors, it corrects the GFI statistic based on the degrees of freedom and the number of variables, and both the GFI and AGFI reach a value of 1 when all residuals are zero [19]. The root mean square error of approximation (RMSEA) values between 0.05 and 0.08 or less indicate a reasonable error of approximation, and values greater than 0.1 indicate that the model is not adequate [20]; and the comparative fit index (CFI), values close to 1 indicate a very good fit [21].

### 2.4. Ethical Aspects

The research presented herein was evaluated and approved regarding its ethical and methodological aspects by researchers of the Social and Health Psychology Research Group of the Autonomous University of Nuevo León (CAPS-20-19-11). At the beginning of the questionnaire, the purpose of the research was explained, and participants were asked to proceed if they willingly agreed to participate. The anonymity and confidentiality of the information provided was guaranteed at all times.

## 3. Results

### 3.1. Personality Traits Based on Cat Coat Color

In this study, 211 people with an average age of 34.3 years (SD = 10.9) participated, 81.0% of whom were female (*n* = 171), while 19.0% were male (*n* = 40). A total of 56.9% of the participants were single, 37.9% were married or in a domestic partnership, 3.8% were divorced or separated, and 1.4% were widowed. The participants indicated that they had an average of 2.9 cats (SD = 3.2, median = 2.0). Overall, 48.8% of the participants reported having at least one dog and one cat. The average age of the cats was 4.7 years (SD = 3.7); 57.0% of the cats were female, and 43.0% were male.

**Figure 1:**
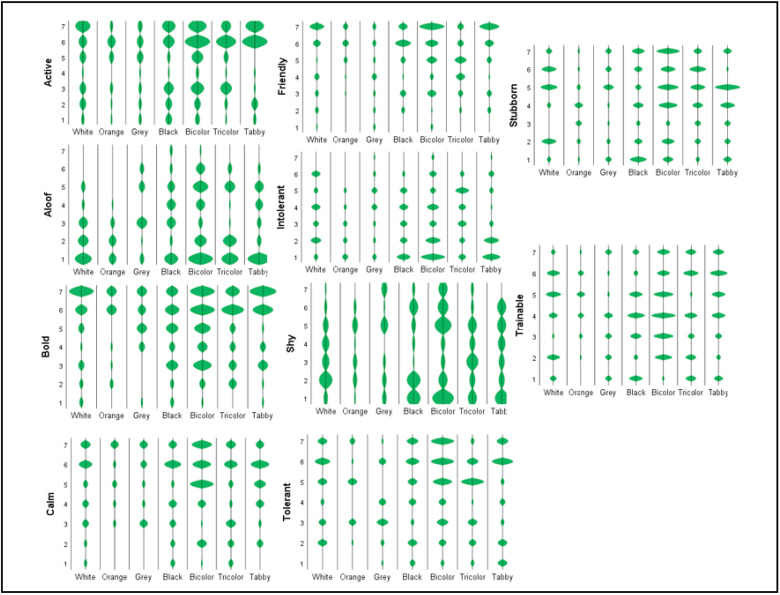
Personality traits based on cat coat color: individual distribution.

Table 1 provides the descriptive statistics and the estimated index for each personality trait based on the coat color of the cat. Figure 1 shows individual distributions of the scores. Most cats were described by their owners as bold and friendly. When comparing the cats by coat color, gray cats had the highest scores for shyness, aloofness and intolerance, while orange cats had the highest scores for trainability, friendliness and calmness. Tabby cats had the highest scores for bold and active, tricolor cats for stubborn, and bicolor cats for tolerant. In each of the personality traits, the mentioned coat color is more than one standard deviation above the sample mean, with the exception of tabby cats in the bold personality trait (Table 1).

Although no significant differences in personality traits (*p* > 0.05) were found when comparing the groups based on coat color. The size of each group is a factor that could explain it. When performing the Kruskal Wallis analysis with weighted data, simulating that each group is 5 times larger, all differences were significant.

### 3.2. Cat-Owner Relationship Scale: Mexican Version

The following results address the psychometric properties of the Mexican version of the CORS. The internal consistency considering all 26 items was 0.84, with adequate inter-item correlations except for items 11 (*r* = −0.054, item: My cat costs too much) and 16 (*r* = 0.017, item: How often does your cat stop you from doing things that you want to do?). When eliminating these items, the alpha increased to 0.85.

For the closeness subscale, the alpha was 0.86, and the inter-item correlations ranged from 0.35 to 0.69. For the interaction subscale, the alpha was 0.80, and the inter-item correlations ranged from 0.47 to 0.63. For the perceived cost subscale, the alpha was 0.74, and the inter-item correlations ranged from 0.29 to 0.56.

Based on the exploratory factor analysis (Table 2) results, items 12 and 13 had high factorial loads in the cat-owner interaction subscale and low factorial loads in the perceived emotional closeness subscale, which is where they belong according to the CORS authors.

A confirmatory factor analysis was performed with 3 correlated factors. The covariance between the cat-owner interaction and perceived cost factors was the only nonsignificant parameter (*p* = 0.690); however, when setting this parameter to zero, the goodness-of-fit statistics still indicated that the model should be improved (X^2^/df = 2.973; GFI = 0.766; AGFI = 0.725; CFI = 0.684; RMSEA = 0.097: CI 0.090–0.104). A second model was tested with items 12 and 13 in the cat-owner interaction subscale, and the only nonsignificant parameter (*p* = 0.561) again corresponded to the same covariance, while the goodness-of-fit statistics indicated improvement in the model (X^2^/df = 2.905; GFI = 0.773; AGFI = 0.733; CFI = 0.695; RMSEA = 0.095: CI 0.088–0.103).

Based on the above, we decided to retain the items in the subscales indicated by Howell et al. [2] and estimate independent models for each subscale, thus obtaining significant parameters and adequate statistical goodness of fit for each subscale: perceived emotional closeness (X^2^/df = 2.475; GFI = 0.913; AGFI = 0.857; CFI = 0.936; RMSEA = 0.084: CI 0.063–0.105); cat-owner interaction (X^2^/df = 2.841; GFI = 0.962; AGFI = 0.912; CFI = 0.949; RMSEA = 0.094: CI 0.052–0.137); and perceived cost (X^2^/df = 2.494; GFI = 0.943; AGFI = 0.897; CFI = 0.897; RMSEA = 0.084: CI 0.058–0.111). Thus, for the following analyses, the subscales were considered independently.

### 3.3. Cat Coat Color and Cat Owner Relationship

**Figure 2:**
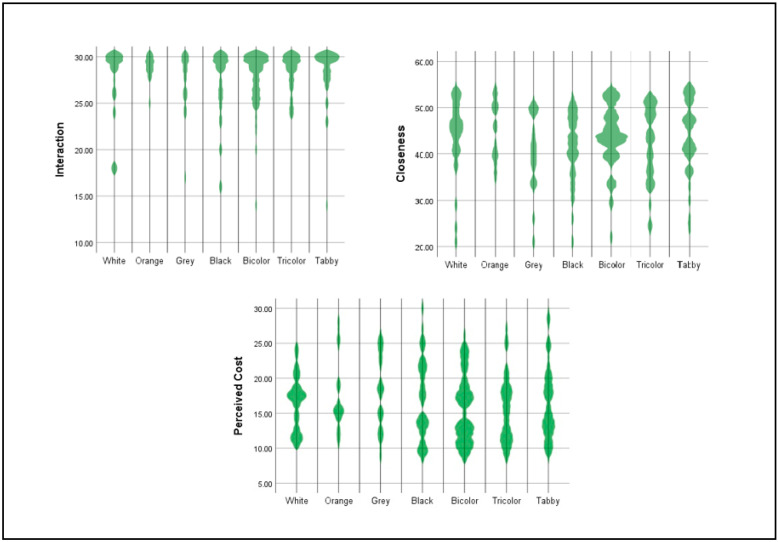
Cat colors and CORS subescales: individual distribution.

The descriptive statistics are presented in Table 3 and individual distribution are showed in Figure 2. A higher score indicates a stronger presence of the variable; that is, a higher score reflects a higher perceived cost, greater emotional closeness and a better interaction. For the sum of the CORS scores, the items for perceived cost were recoded. The means are also shown because these data are usually reported in the MDORS. Based on these data, the participants presented high levels of interaction and closeness with their cats and perceived the cost of their relationships to be low.

Considering the groups based on coat color, the differences in the interaction (H = 5.593; *p* = 0.470) and closeness (H = 7.623; *p* = 0.267) scores were analyzed using Kruskal-Wallis test. Although orange cats scored highest for interaction and closeness, the differences among cats with different coat colors were not significant. Additionally, no significant differences in perceived cost (H = 4.104; *p* = 0.663) were found when comparing the groups based on coat color.

### 3.4. Personality Traits and Cat Owner Relationship

Table 4 shows the correlations between the personality traits of cats, and between personality traits and the CORS subscales. The cat-owner interaction subscale was positively correlated with the active and friendly traits and negatively correlated with aloofness. Emotional closeness was positively correlated with activeness, boldness and friendliness, which were predominant in the cats (see Table 1). Perceived cost was negatively correlated with bold cats. Nevertheless, some of these correlations were weak (*r* < 0.20). The other predominant trait, calmness, was not correlated with the CORS.

### 3.5. Other Variables

Other variables could explain the differences in personality traits among cats and the cat-owner relationship, the analyzes presented below were performed after the fact, we included age and sex of the cats, and age and gender of the owner.

#### 3.5.1. Cat Age

Correlation between personality traits and cat age was analyzed. Negative relations were found between age and active (*r_s_* = −0.249; *p* = 0.001), bold (*r_s_* = −0.146; *p* = 0.034) and trainable (*r_s_* = −0.211; *p* = 0.002). And positive related with calm (*r_s_* = 0.167; *p* = 0.015). Other correlation with age were no significant (*p* > 0.05). Thus, age of the cats could explain some personality traits, a structural equation model was performed to analyzed it (Table 5) and low percentage of explained variance was found for each personality trait; which is consistent with the correlation coefficients that were mostly weak.

Age of the cats was equivalent among the cat color groups (H = 2.920; *p* = 0.819), thus age does not explain results showed in Table 1.

The correlations between age and the CORS subscales were analyzed. Emotional closeness was positively and weak correlated with age (*r_s_* = 0.170; *p* = 0.014). The cat-owner interaction subscale (*r_s_* = −0.014; *p* = 0.843) and perceived cost (*r_s_* = −0.115; *p* = 0.097) were no significantly related with cat age.

#### 3.5.2. Cat Sex

**Figure 3:**
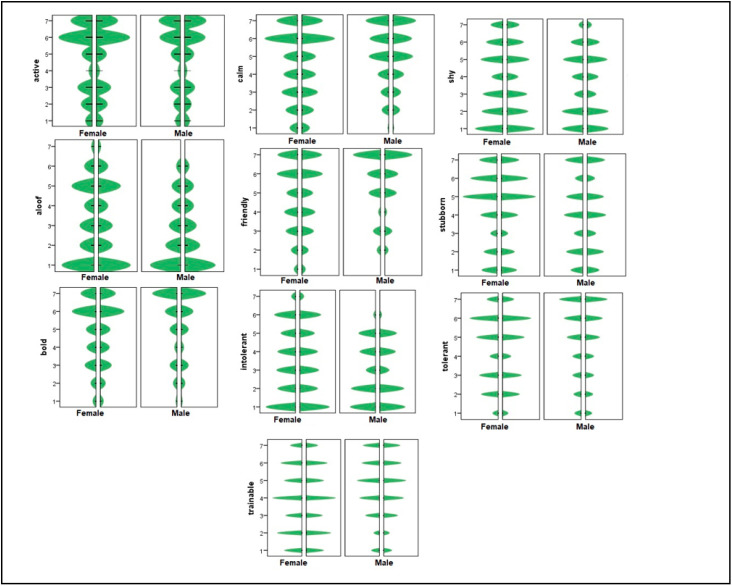
Personality traits and cat sex: individual distribution.

Differences between male and female cats regarding personality traits were analyzed, significant differences were found in aloof, bold, friendly, intolerant and trainable. Thus, sex of the cat could explain personality traits. Figure 3 shows individual distributions of the scores.

Although the distribution of cats according to sex is equivalent in the complete sample (57.0% female, 43.0% male), in the groups by cat color there is a predominance of males in orange cats and females in gray, tricolor and tabby. In addition, color and sex are significantly associated (X^2^ = 22.791; *p* = 0.001).

We analyze differences in personality traits separating male and female cats, no significant differences among color cat groups (*p* > 0.05) were found. Analyzing the differences in personality between males and females, separating by colors, significant differences were only found in white cats in these personality traits: aloof, bold, friendly, intolerant, stubborn and tolerant (*p* < 0.05) (Table 6).

Higher score for male cats were found in emotional closeness (*Z* = 2.288; *p* = 0.022). No significant differences were found between male and female cats when the cat-owner interaction subscale (*Z* = −1.625; *p* = 0.104) and perceived cost (*Z* = −0.278; *p* = 0.781) were analyzed.

#### 3.5.3. Owner Age

Personality traits is not an objective variable, it reflects owners’ perception, therefor some owner variables, such as age and gender, could explain it. Correlation between personality traits and owner age were no significant (*p* > 0.05). Separating cats according its color and comparing owner age among groups we found age of the owner was equivalent among the groups (H = 8.451; *p* = 0.207). These results suggest owners’ age is not a variable that could explain their perception of personality traits. Nevertheless, a structural equation model was performed to analyzed it. All regression weights for owner age in the prediction of each personality trait were not significant (*p* > 0.05).

The correlations between owner age and the CORS subscales were analyzed. Emotional closeness (*r_s_* = 0.200; *p* = 0.004) and perceived cost (*r_s_* = −0.165; *p* = 0.017) were significantly correlated with owner age. The cat-owner interaction subscale (*r_s_* = 0.090; *p* = 0.195) was no significantly related with owner age.

#### 3.5.4. Owner Gender

Most of participants were female (*n* = 171, 81.0%). We found equivalence in scores of each personality trait between perception of male and female owners (*p* > 0.05).

When analyzing the differences in the perception of personality traits, separating men and women, the perception of men indicates significant differences exclusively in stubborn personality when comparing cats according to their color (*p* < 0.05). Tricolor cats had highest score in complete sample, men and women groups (Table 7). Color with the highest score in each personality trait does not change when men are excluded from analysis; nevertheless, considering only men, color with the highest score in each personality trait varies.

Significant differences between men and women were found comparing scores of emotional closeness (*Z* = 3.691; *p* = 0.001) and cat-owner interaction subscale (*Z* = −2.066; *p* = 0.039), with higher scores from women. Perceived cost scores were equivalents (*Z* = −1.766; *p* = 0.77).

## 4. Discussion

In this study, people reported to perceive their cats as bold and friendly. Highest score for each personality trait showed that gray cats had the highest score for being as shy, aloof and intolerant, while orange cats had the highest scores for being trainable, friendly and calm. Tabbies the highest for bold and active, tricolor cats for stubborn, and bicolor cats for tolerant. No significant differences in personality traits (*p* > 0.05) were found when comparing the groups based on coat color. It is possible that with a larger sample size and balanced groups in size, the differences were significant.

Contextualizing the findings of any coat color study with the existing literature is not simple due to the lack of uniform color patterning in cats [12]. The results of the present study partially coincide with those found by Delgado et al. [10], who reported that orange cats are considered friendly and tricolor cats are intolerant. Nevertheless, Stelow et al. [6] indicated that recent studies found no relationship between cat coat color and behavior.

Notably, despite the myths surrounding black cats, their predominant trait according to the owners’ reports is friendliness. Jones & Hart [22] using evaluation of cats’ photos found in their sample that people rated black cats as less friendly and more aggressive than their non-black counterparts. For dogs, anecdotal reports indicate that black dogs take longer to be adopted; however, Díaz Videla [8] found that color is not a useful predictor of the fate of shelter dogs. Jones & Hart [22] did not find a bias related to black cats in the willingness to adopt cats. These studies are evidence that people’s perception of cats may differ from their own cat’s personality perception. Therefore, it is necessary to conduct more researches such as the present one, in which the perception of the own cat is evaluated.

Physiological aspects must be considered in the study of the variables related to the cat’s personality. As mentioned above, different studies have showed evidence of the relationship between the physical appearance of mammals and personality traits [7,8], which is based on the fact that the pigment melanin shares a synthesis pathway with a group of catecholamines and neurotransmitters [9], such as dopamine, which may lead to associations between pigmentation and personality traits [8]. Personality trait is a multidetermined variable. Other variables, in addition to the color of the cats, could explain the differences in personality traits. The age and sex of the cats, as well as the age and gender of the owners, were analyzed. The age of cats is logically related to personality traits such as active, trainable and bold. In our study, the age of the cats was equivalent among the groups according to the color of the cats. The results showed that the sex of cats is associated with their color. There is a predominance of males in orange cats and females in gray, tricolor and tabby. These colors are the ones that obtained the highest scores in most personality traits. Sex of the cat may be an important variable in the study of cat personality.

Owner variables could affect their perception of personality traits in their cats, we analyzed age and gender of the owner. The highest percentage of the sample corresponds to women, so it was analyzed whether the difference in some scores when comparing men and women affects our conclusions, when performing the analysis exclusively with women, the colors identified with higher scores in each trait of personality does not vary. However, it is detected that the perception of men and women can vary, for future research it is suggested that men and women of the same family be evaluated to compare their perception of the predominant personality trait in the same cat.

A good owner-cat relationship can provide what is known as the pet effect, which refers to the benefits to an individual’s emotional well-being in the presence of a pet [23]. Identifying factors associated with a good owner-cat relationship, such as cats’ personality traits, will allow us to further elucidate the pet effect. According to the results reported by Janssens et al. [23], this effect varies between the presence of and interaction with a companion animal. When a pet is simply present (passive), negative feelings are reduced; however, interacting with the pet generates positive feelings.

The cat-owner interaction was found to be positively correlated with the traits: activeness and friendliness and negatively correlated with aloofness. Based on this result and the findings of Janssens et al. [23], an emotional benefit to humans can be reasonably inferred from this interaction. Likewise, emotional closeness was positively correlated with active, bold and friendly traits. Perceived cost was negatively correlated with boldness, implying that the negative consequences of having a cat are less important to the owner with increasing boldness of the cat; however, the opposite trait, calmness, was not correlated with the CORS. Active and friendly cats seem to have the best relationships with humans. Age and sex of the cat could be factors associated with a good cat-owner relationship, we found higher score for male cats in emotional closeness, and a positive correlation between emotional closeness and cat age. This correlation is lower than 0.20, thus this correlation is considered weak. More research is needed including these and other variables to identify factors associated with a good cat-owner relationship and with the pet effect.

Interestingly, the trainability trait was significantly correlated with the CORS and its positive subscales, implying that this trait is important to owners; it reflects the need for cat trainers, which is not common in Mexico.

Although no significant differences were detected in the CORS subscales, orange cats had the highest scores for the traits trainability, friendliness and calmness as well as for the interaction and emotional closeness subscales of the CORS, thus supporting the hypothesis that these cats have the best relationships with their owners, which should be tested in future research.

Owner variables were related to CORS subescales, age of the owner was significantly correlated with emotional closeness and perceived cost; while women had higher scores in emotional closeness and cat-owner interaction than men. These results indicate that both parts of the relationship, cat and owner, should be considered to explain the factors associated with a good human-cat relationship.

Regarding the translation of the CORS, the scores for each subscale should be considered independently, as in the present study; accordingly, the 3 scales have adequate psychometric properties. An important contribution to the field of study is our Spanish version of the CORS, which will allow an increase in research on the cat-owner relationship in Spanish.

Among the limitations of the study are that most of the participants were women, which is common in these studies. Small sample size is another limitation; 69.8% of households in Mexico have a pet, almost 80 million pets live in these households, of which 16.2 million are cats [24] and we only had information about 211 cats. Other variables not considered in this study may have influenced the participants’ perceptions, variables such as the breed, desexing status, if the cat was adopted, rescued, or bought; the reasons why the particular cat was selected to be part of the family, cat color could be a reason. Additionally, high scores on the CORS suggest a good cat-owner relationship, which is another common element with respect to voluntary participation in studies of human-animal bonds, implying biased results.

In addition, color classifications are prone to errors. Carini et al. [12] indicated that coat color in cats is difficult to describe due to the wide variety of patterns that exist. Therefore, color assessment is subjective and results in incongruity between the findings of different studies on the subject. In future research, we suggest that a photograph of each cat be provided by the owner and that the team of researchers, not the owners, classify the coat color.

## 5. Conclusions

It is concluded that perceived personality traits could be related to the sex and coat colors of cats, and these traits are one of the factors related to the interaction, emotional closeness and perceived cost subscales of the CORS. However, these results are based on the owners’ perceptions and they are not the product of empirical observations.

Further studies with larger samples are necessary to provide more research evidence regarding the cat-owner bond.

## Figures and Tables

**Table 1:** Personality traits based on cat coat color.

	Active	Aloof	Bold	Calm	Friendly	Intolerant	Shy	Stubborn	Tolerant	Trainable	Predominant Trait
	White cat (*n* = 29)										
Mean	4.8	2.3	5.3	4.8	5.2	3.6	3.3	4.4	4.8	4.1	Friendly and bold
Median	5.0	2.0	6.0	5.0	6.0	4.0	3.0	5.0	5.0	5.0	
SD	2.1	1.3	2.0	1.9	2.0	1.7	1.6	1.9	1.8	1.9	
Index	68.5	32.5	75.9	68.5	73.9	51.2	46.8	62.6	68.0	58.6	
	Yellow or orange cat (*n* = 16)										
Mean	4.9	2.0	5.4	5.4	5.7	2.6	3.7	3.8	4.7	4.6	Friendly, calm and bold
Median	5.5	2.0	6.0	5.5	6.0	2.5	3.5	4.0	5.0	5.0	
SD	1.9	1.0	1.9	1.5	1.2	1.4	1.6	1.6	1.7	1.4	
Index	69.4	28.6	77.6	77.6	81.6	37.8	53.1	54.1	67.3	66.3	
	Gray cat (*n* = 18)										
Mean	4.5	3.6	5.4	5.1	4.3	3.8	4.1	4.3	3.9	3.7	Bold and calm
Median	5.0	3.0	5.5	5.5	4.0	4.0	5.0	5.0	3.5	3.5	
SD	2.0	1.7	1.5	1.6	2.0	1.8	2.2	2.1	1.7	2.1	
Index	64.3	50.8	77.0	73.0	61.1	54.0	58.7	61.9	55.6	52.4	
	Black cat (*n* = 32)										
Mean	4.4	3.3	4.7	4.6	5.2	3.0	3.2	3.8	4.5	3.8	Friendly
Median	5.0	3.0	5.0	5.0	6.0	3.0	2.0	4.0	5.0	4.0	
SD	2.2	1.9	1.8	2.0	1.7	1.7	2.2	2.3	2.0	1.8	
Index	63.4	47.3	67.0	65.2	74.1	42.4	46.0	54.9	64.3	53.6	
	Bicolor cat (*n* = 50)										
Mean	4.9	3.2	5.0	5.1	5.4	2.9	3.7	4.6	5.0	4.2	Friendly
Median	6.0	3.0	5.0	5.0	6.0	2.0	4.0	5.0	5.0	4.0	
SD	1.9	2.0	1.7	1.7	1.7	1.9	2.2	2.0	1.8	1.7	
Index	70.0	45.1	71.4	73.1	77.1	42.0	53.1	65.1	71.4	60.3	
	Tricolor cat (*n* = 32)										
Mean	5.0	2.8	5.1	4.4	4.8	3.6	3.4	4.6	4.3	4.1	Bold and active
Median	6.0	2.0	6.0	5.0	5.0	4.0	3.0	5.0	5.0	4.0	
SD	1.8	1.9	1.7	2.1	1.4	1.6	1.8	1.8	1.7	2.1	
Index	71.0	39.7	72.3	63.4	68.8	50.9	49.1	66.1	62.1	58.0	
	Tabby cat (*n* = 34)										
Mean	5.4	2.7	5.5	5.0	5.6	2.7	3.4	4.1	4.4	4.5	Friendly and bold
Median	6.0	2.0	6.0	5.0	6.0	2.0	3.5	4.5	5.5	5.0	
SD	2.0	1.8	1.8	1.6	1.7	1.9	1.9	1.8	2.2	1.9	
Index	77.3	38.7	78.2	71.4	79.4	38.2	48.3	59.2	63.4	64.7	
Kruskal-Wallis test	H = 5.130 *p* = 0.527	H = 11.000 *p* = 0.0.88	H = 6.200 *p* = 0.401	H = 4.075 *p* = 0.667	H = 10.956 *p* = 0.090	H = 11.257 *p* = 0.081	H = 3.152 *p* = 0.789	H = 5.125 *p* = 0.528	H = 6.344 *p* = 0.386	H = 5.324 *p* = 0.503	
Weighted data	H = 25.750 *p* = 0.001	H = 56.211 *p* = 0.001	H = 31.120 *p* = 0.001	H = 20.454 *p* = 0.002	H = 55.007 *p* = 0.001	H = 56.503 *p* = 0.001	H = 15.823 *p* = 0.015	H = 25.722 *p* = 0.001	H = 31.840 *p* = 0.001	H = 26.722 *p* = 0.001	
Highest * *Z*-score	Tabby 1.78	Gray 1.30	Tabby 0.97	Orange 1.48	Orange 1.14	Gray 1.32	Gray 1.77	Tricolor 1.18	Bicolor 1.35	Orange 1.38	

SD: standard deviation; *n*: number of cats in this group; index: the extent to which the personality trait is evident in this coat color group; * Color with the highest score in each personality trait.

**Table 2:** Exploratory factor analysis for the CORS.

	Perceived Emotional Closeness	Cat-Owner Interaction	Perceived Cost
(7) ¿Con que frecuencia juegas con tu gato? (How often do you play games with your cat?)		0.598	
(9) ¿Con qué frecuencia disfrutas del tiempo tan solo viendo a tu gato? (How often do you spend time enjoying watching your cat?)		0.638	
(15) ¿Con que frecuencia le hablas a tu gato? (How often do you talk to your cat?)		0.608	
(21) ¿Con que frecuencia cargas o tienes en tus brazos a tu gato? (How often do you cuddle your cat?)		0.787	
(23) ¿Con que frecuencia tu gato está contigo mientras te relajas (por ejemplo mientras ves televisión)? (How often do you have your cat with you while relaxing, e.g., watching TV?)		0.641	
(26) ¿Con que frecuencia acaricias a tu gato? (How often do you pet your cat?)		0.758	
(2) Mi gato me da una razón para levantarme por la mañana. (My cat gives me a reason to get up in the morning.)	0.606		
(4) ¿Con que frecuencia le das besos a tu gato? (How often do you kiss your cat?)	0.401	0.593	
(5) Quisiera que mi gato y yo nunca tuviéramos que separarnos. (I wish my cat and I never had to be apart.)	0.723		
(12) ¿Con que frecuencia compras cosas para tu gato (regalos, juguetes, etc.) (How often do you buy your cat presents?		0.400	
(13) ¿Con que frecuencia le dices cosas a tu gato que no le dices a nadie mas? (How often do you tell your cat things that you do not tell anyone else?)	0.284	0.402	
(17) Quisiera que mi gato estuviera conmigo todo el tiempo. (I would like to have my cat near me all the time.)	0.714		
(18) Si todos me dejaran, mi gato estaría ahí para mí (contaría con mi gato). (If everyone else left me, my cat would still be there for me.)	0.767		
(20) Mi gato me ayuda en tiempos difíciles. (My cat helps me get through tough times.)	0.803		
(22) Mi gato me brinda compañía constante. (My cat provides me with constant companionship.)	0.734	0.252	
(24) Mi gato siempre está para mí cuando necesito consuelo. (My cat is there whenever I need to be comforted.)	0.798		
(25) ¿Qué tan doloroso crees que será para ti cuando tu gato muera? (How traumatic do you think it will be for you when your cat dies?)	0.569		
(1) ¿Qué tan difícil es cuidar de tu gato? (How hard is it to look after your cat?)			0.705
(3) Hay cosas importantes relacionadas a tener a mi gato que no me gustan. (There are major aspects of owning a cat that I do not like.)			0.615
(6) Mi gato hace mucho desorden. (My cat makes too much mess.)			0.391
(8) Me molesta que por mi gato he dejado de hacer cosas que disfrutaba haciendo antes de tenerlo. (It bothers me that my cat stops me from doing things that I enjoyed before I owned it.)			0.657
(10) Es molesto que algunas veces tengo que cambiar mis planes debido a mi gato. (It is annoying that I sometimes have to change my plans because of my cat.)			0.691
(11) Gasto mucho dinero en mi gato. (My cat costs too much money.)			0.414
(14) ¿Con que frecuencia sientes que cuidar a tu gato es un trabajo difícil? (How often do you feel that looking after your cat is a chore?)			0.561
(16) ¿Con que frecuencia tu gato te limita de hacer cosas que tú quieres hacer? (How often does your cat stop you from doing things that you want to do?)			0.606
(19) ¿Con que frecuencia sientes que tener gato es más costo que beneficio? (How often do you feel that having a cat is more trouble than it is worth?)			0.443
Variance explained	24.1%	12.5%	7.9%
Cronbach’s alpha	0.86	0.80	0.74

Factorial loads higher than 0.40 are shown, and also those for items 12 and 13 in the subscale where they belong according to the CORS authors.

**Table 3:** Descriptive statistics for the CORS.

	Mean	Median	Standard Deviation	K-S Normality Test
Cat-owner interaction	27.7	29.0	3.3	*Z* = 0.244; *p* = 0.001
Mean cat-owner interaction	4.6	4.8	0.5	
White cat	4.5	4.8	0.7	
Orange cat	4.8	4.8	0.2	
Gray cat	4.5	4.7	0.6	
Black cat	4.4	4.8	0.7	
Bicolor cat	4.6	4.8	0.5	
Tricolor cat	4.7	4.8	0.3	
Tabby cat	4.7	4.9	0.5	
Perceived emotional closeness	42.9	43	7.8	*Z* = 0.089; *p* = 0.001
Mean perceived emotional closeness	3.9	3.9	0.7	
White cat	4.0	4.2	0.7	
Orange cat	4.1	4.2	0.6	
Gray cat	3.6	3.6	0.8	
Black cat	3.7	3.8	0.7	
Bicolor cat	3.9	3.9	0.6	
Tricolor cat	3.8	3.9	0.7	
Tabby cat	3.9	4.0	0.7	
Perceived cost	16.6	17.0	5.0	*Z* = 0.111; *p* = 0.001
Mean perceived cost	1.8	1.9	0.6	
White cat	1.8	1.9	0.4	
Orange cat	1.9	1.7	0.6	
Gray cat	2.0	2.0	0.6	
Black cat	1.9	1.9	0.6	
Bicolor cat	1.7	1.7	0.5	
Tricolor cat	1.7	1.7	0.5	
Tabby cat	1.8	1.7	0.6	
Mean CORS scores	4.1	4.2	0.4	*Z* = 0.081; *p* = 0.002
White cat	4.2	4.2	0.4	
Orange cat	4.2	4.2	0.4	
Gray cat	4.0	4.0	0.4	
Black cat	4.0	4.1	0.4	
Bicolor cat	4.2	4.2	0.4	
Tricolor cat	4.2	4.1	0.4	
Tabby cat	4.2	4.3	0.5	

K-S: Kolmogorov-Smirnov test.

**Table 4:** Spearman correlations between CORS subscales and personality traits.

	1	2	3	4	5	6	7	8	9	10	11	12
(1) Cat-owner interaction	1											
(2) Perceived emotional closeness	0.576 **	1										
(3) Perceived cost	−0.026	−0.104	1									
(4) Active	0.225 **	0.166 *	−0.120	1								
(5) Aloof	−0.280 **	−0.133	0.117	−0.047	1							
(6) Bold	0.122	0.153 *	−0.197 **	0.544 **	0.004	1						
(7) Calm	−0.084	0.049	−0.096	−0.401 **	0.054	−0.237 **	1					
(8) Friendly	0.184 **	0.146 *	−0.089	0.090	−0.498 **	0.069	0.144 *	1				
(9) Intolerant	−0.176*	−0.074	0.094	−0.026	0.400 **	0.026	−0.109	−0.551 **	1			
(10) Shy	0.009	0.027	−0.002	−0.063	0.139 *	−0.169 *	0.205 **	−0.293 **	0.214 **	1		
(11) Stubborn	0.058	0.105	0.074	0.024	0.172 *	0.026	−0.057	−0.275 **	0.384 **	0.106	1	
(12) Tolerant	0.144 *	0.065	−0.083	0.179 **	−0.376 **	0.096	0.144 *	0.569 **	−0.568 **	−0.204 **	−0.182 **	1
(13) Trainable	0.313 **	0.199 **	−0.052	0.291 **	−0.345 **	0.216 **	−0.044	0.369 **	−0.291 **	−0.071	−0.047	0.413 **

* *p* < 0.05; ** *p* < 0.01.

**Table 5:** Personality traits and cat age.

			S	*p*	Explained Variance
Active	⇦	Cat age	−0.287	0.001	8.2%
Aloof	⇦	Cat age	−0.047	0.494	0.2%
Bold	⇦	Cat age	−0.217	0.001	4.7%
Calm	⇦	Cat age	0.133	0.051	1.8%
Friendly	⇦	Cat age	−0.126	0.065	1.6%
Intolerant	⇦	Cat age	0.104	0.128	1.1%
Shy	⇦	Cat age	−0.015	0.826	0.0%
Stubborn	⇦	Cat age	0.067	0.128	1.1%
Tolerant	⇦	Cat age	−0.106	0.122	1.1%
Trainable	⇦	Cat age	−0.268	0.001	7.2%

S = Standardized estimates; *p* = Statistical significance.

**Table 6:** Personality traits, cat sex and cat coat color.

	Active	Aloof	Bold	Calm	Friendly	Intolerant	Shy	Stubborn	Tolerant	Trainable
Mean (SD) male	4.9 (1.9)	2.5 (1.6)	5.4 (1.8)	5.1 (1.6)	5.6 (1.6)	2.8 (1.5)	3.4 (1.9)	4.1 (2.0)	4.8 (1.9)	4.5 (1.8)
Mean (SD) female	4.8 (1.9)	3.1 (1.9)	4.9 (1.8)	4.7 (1.9)	4.9 (1.8)	3.4 (1.9)	3.6 (2.0)	4.5 (1.9)	4.4 (1.8)	3.8 (1.8)
Male vs. female	*Z* = −0.703; *p* = 0.482	*Z* = −2.199; *p* = 0.028	*Z* = −2.607; *p* = 0.009	*Z* = −1.645; *p* = 0.100	*Z* = −3.059; *p* = 0.002	*Z* = −2.186; *p* = 0.029	*Z* = −0.579; *p* = 0.563	*Z* = −1.527; *p* = 0.127	*Z* = −1.936: *p* = 0.053	*Z* = −2.709; *p* = 0.007
Color with the highest score in each personality trait										
	Tabby	Gray	Tabby	Orange	Orange	Gray	Gray	Tricolor	Bicolor	Orange
% male % female	39.4% 60.6%	27.8% 72.2%	39.4% 60.6%	78.6% 21.4%	78.6% 21.4%	27.8% 72.2%	27.8% 72.2%	15.6% 84.4%	55.1% 44.9%	78.6% 21.4%

**Table 7:** Owner gender, personality traits and cat coat color.

Owner Gender	Active	Aloof	Bold	Calm	Friendly	Intolerant	Shy	Stubborn	Tolerant	Trainable
Male	*Z* = 10.141; *p* = 0.119	*Z* = 5.579; *p* = 0.472	*Z* = 10.129; *p* = 0.119	*Z* = 12.129; *p* = 0.059	*Z* = 6.233; *p* = 0.398	*Z* = 3.589; *p* = 0.732	*Z* = 4.663; *p* = 0.588	*Z* = 13.667; *p* = 0.034	*Z* = 4.299; *p* = 0.636	*Z* = 12.818; *p* = 0.046
Female	*Z* = 9.393; *p* = 0.153	*Z* = 7.981; *p* = 0.240	*Z* = 7.228; *p* = 0.300	*Z* = 8.979; *p* = 0.175	*Z* = 7.148; *p* = 0.307	*Z* = 8.192; *p* = 0.224	*Z* = 4.698; *p* = 0.583	*Z* = 1.436; *p* = 0.964	*Z* = 6.855; *p* = 0.335	*Z* = 4.501; *p* = 0.609
Color with the highest score in each personality trait										
Total sample	Tabby	Gray	Tabby	Orange	Orange	Gray	Gray	Tricolor	Bicolor	Orange
Male owners	Black	Black	Orange	White	Orange	Gray	White	Tricolor	White	Tabby
Female owners	Tabby	Gray	Tabby	Orange	Orange	Gray	Gray	Tricolor	Bicolor	Orange

## Data Availability

The data supporting the findings of this study are available upon request from the corresponding author.

## Appendix A

**Table A1:** Cat Owner Relationship Scale—Mexican Version (CORS-M).

Instrucciones: considera cada una de las siguientes afirmaciones e indica qué opción describe mejor cómo te sientes o actúas. Estamos interesados en tus opiniones. No hay respuestas correctas o incorrectas.					
1. ¿Qué tan difícil es cuidar de tu gato?	Muy difícil	Difícil	Ni fácil ni difícil	Fácil	Muy fácil
2. Mi gato me da una razón para levantarme por la mañana.	Totalmente de acuerdo	De acuerdo	Ni en acuerdo ni en desacuerdo	En desacuerdo	Totalmente en desacuerdo
3. Hay cosas importantes relacionadas a tener a mi gato que no me gustan	Totalmente de acuerdo	De acuerdo	Ni en acuerdo ni en desacuerdo	En desacuerdo	Totalmente en desacuerdo
4. ¿Con que frecuencia le das besos a tu gato?	Por lo menos una vez al día	Algunos días de la semana	Una vez a la semana	Una vez al mes	Nunca
5. Quisiera que mi gato y yo nunca tuviéramos que separarnos	Totalmente de acuerdo	De acuerdo	Ni en acuerdo ni en desacuerdo	En desacuerdo	Totalmente en desacuerdo
6. Mi gato hace mucho desorden	Totalmente de acuerdo	De acuerdo	Ni en acuerdo ni en desacuerdo	En desacuerdo	Totalmente en desacuerdo
7. ¿Con que frecuencia juegas con tu gato?	Por lo menos una vez al día	Algunos días de la semana	Una vez a la semana	Una vez al mes	Nunca
8. Me molesta que por mi gato he dejado de hacer cosas que disfrutaba haciendo antes de tenerla	Totalmente de acuerdo	De acuerdo	Ni en acuerdo ni en desacuerdo	En desacuerdo	Totalmente en desacuerdo
9. ¿Con qué frecuencia disfrutas del tiempo tan solo viendo a tu gato?	Al menos una vez al día	Una vez a la semana	Una vez al mes	Un par de veces al año	Nunca
10. Es molesto que algunas veces tengo que cambiar mis planes debido a mi gato	Totalmente de acuerdo	De acuerdo	Ni en acuerdo ni en desacuerdo	En desacuerdo	Totalmente en desacuerdo
11. Gasto mucho dinero en mi gato	Totalmente de acuerdo	De acuerdo	Ni en acuerdo ni en desacuerdo	En desacuerdo	Totalmente en desacuerdo
12. ¿Con que frecuencia compras cosas para tu gato (regalos, juguetes, etc.)	Una vez a la semana	Una vez cada dos semanas (cada quincena)	Una vez al mes	Un par de veces al año	Nunca
13. ¿Con que frecuencia le dices cosas a tu gato que no le dices a nadie mas?	Una vez al día	Una vez a la semana	Una vez al mes	Una vez al año	Nunca
14. ¿Con que frecuencia sientes que cuidar a tu gato es un trabajo difícil?	Una vez al día	Una vez a la semana	Una vez al mes	Una vez al año	Nunca
15. ¿Con que frecuencia le hablas a tu gato?	Al menos una vez al día	Algunos días de la semana	Una vez a la semana	Una vez al mes	Nunca
16. ¿Con que frecuencia tu gato te limita de hacer cosas que tú quieres hacer?	Una vez al día	Una vez a la semana	Una vez al mes	Una vez al año	Nunca
17. Quisiera que mi gato estuviera conmigo todo el tiempo	Totalmente de acuerdo	De acuerdo	Ni en acuerdo ni en desacuerdo	En desacuerdo	Totalmente en desacuerdo
18. Si todos me dejaran, mi gato estaría ahí para mí (contaría mi gato)	Totalmente de acuerdo	De acuerdo	Ni en acuerdo ni en desacuerdo	En desacuerdo	Totalmente en desacuerdo
19. ¿Con que frecuencia sientes que tener gato es más costo que beneficio?	Una vez al día	Una vez a la semana	Una vez al mes	Una vez al año	Nunca
20. Mi gato me ayuda en tiempos difíciles	Totalmente de acuerdo	De acuerdo	Ni en acuerdo ni en desacuerdo	En desacuerdo	Totalmente en desacuerdo
21. ¿Con que frecuencia cargas o tienes en tus brazos a tu gato?	Al menos una vez al día	Algunos días de la semana	Una vez a la semana	Una vez al mes	Nunca
22. Mi gato me brinda compañía constante	Totalmente de acuerdo	De acuerdo	Ni en acuerdo ni en desacuerdo	En desacuerdo	Totalmente en desacuerdo
23. ¿Con que frecuencia tu gato está contigo mientras te relajas (por ejemplo mientras ves televisión)?	Al menos una vez al día	Algunos días de la semana	Una vez a la semana	Una vez al mes	Nunca
24. Mi gato siempre está para mí cuando necesito consuelo	Totalmente de acuerdo	De acuerdo	Ni en acuerdo ni en desacuerdo	En desacuerdo	Totalmente en desacuerdo
25. ¿Qué tan doloroso crees que será para ti cuando tu gato muera?	Muy doloroso	Doloroso	Regular	Poco doloroso	Nada doloroso
26. ¿Con que frecuencia acaricias a tu gato?	Al menos una vez al día	Algunos días de la semana	Una vez a la semana	Una vez al mes	Nunca

Scoring instructions for the CORS The CORS consists of three subscales: cat-owner interaction, perceived emotional closeness, and perceived cost. Each item is scored on a five-point scale ranging from 1 to 5. A higher score indicates better perceived relationship quality. To calculate the score for the cat-owner interaction subscale, add the scores for items 7, 9, 15, 21, 23 and 26. Then, divide by 6. To calculate the score for the perceived emotional closeness subscale, add the scores for items 2, 4, 5, 12, 13, 17, 18, 20, 22, 24 and 25. Then, divide by 11. To calculate the perceived cost subscale, add the scores for items 1, 3, 6, 8, 10, 11, 14, 16 and 19. Then, divide by 9.

CORS original version could be found at https://doi.org/10.1016/j.beproc.2017.02.024 (accessed on 5 January 2019)

## Appendix B

**Table A2:** Questionnaire about the Personality Traits of Cats (Spanish Version).

Instrucciones: Indica que tanto cada una de las siguientes características describen a tu gato.							
**Característica**	**Totalmente en Desacuerdo**	**Muy en Desacuerdo**	**Levente en Desacuerdo**	**Ni en Acuerdo ni en Desacuerdo**	**Levemente de Acuerdo**	**Muy de Acuerdo**	**Totalmente de Acuerdo**
Activo (Active)	1	2	3	4	5	6	7
Distante (Aloof)	1	2	3	4	5	6	7
Audaz (Bold)	1	2	3	4	5	6	7
Calmado (Calm)	1	2	3	4	5	6	7
Amigable (Friendly)	1	2	3	4	5	6	7
Intolerante (Intolerant)	1	2	3	4	5	6	7
Tímido (Shy)	1	2	3	4	5	6	7
Obstinado (Stubborn)	1	2	3	4	5	6	7
Tolerante (Tolerant)	1	2	3	4	5	6	7
Entrenable (Trainable)	1	2	3	4	5	6	7
